# Human disturbance and stage-specific habitat requirements influence snowy plover site occupancy during the breeding season

**DOI:** 10.1002/ece3.511

**Published:** 2013-02-28

**Authors:** Alyson F Webber, Julie A Heath, Richard A Fischer

**Affiliations:** 1 Department of Biological Sciences, Boise State UniversityBoise, Idaho, 83725, USA; 2 USACE Engineer Research and Development Center (ERDC), Environmental LaboratoryVicksburg, Mississippi, USA

**Keywords:** Beach, coastal engineering, multi-season occupancy models, nesting, restoration, shorebird

## Abstract

Habitat use has important consequences for avian reproductive success and survival. In coastal areas with recreational activity, human disturbance may limit use of otherwise suitable habitat. Snowy plovers *Charadrius nivosus* have a patchy breeding distribution along the coastal areas on the Florida Panhandle, USA. Our goal was to determine the relative effects of seasonal human disturbance and habitat requirements on snowy plover habitat use. We surveyed 303 sites for snowy plovers, human disturbance, and habitat features between January and July 2009 and 2010. We made multiple visits during three different sampling periods that corresponded to snowy plover breeding: pre-breeding, incubation, and brood-rearing and used multi-season occupancy models to examine whether human disturbance, habitat features, or both influenced site occupancy, colonization (probability of transition from an unoccupied site to an occupied site), and extinction (probability of transition from an occupied site to an unoccupied site). Snowy plover site occupancy and colonization was negatively associated with human disturbance and site extinction was positively associated with human disturbance. Interdune vegetation had a negative effect on occupancy and colonization, indicating that plovers were less likely to use areas with uniform, dense vegetation among dunes. Also, dune shape, beach debris, and access to low-energy foraging areas influenced site occupancy, colonization, and extinction. Plovers used habitat based on beach characteristics that provided stage-specific resource needs; however, human disturbance was the strongest predictor of site occupancy. In addition, vegetation plantings used to enhance dune rehabilitation may negatively impact plover site occupancy. Management actions that decrease human disturbance, such as symbolic fencing and signage, may increase the amount of breeding habitat available to snowy plovers on the Florida Panhandle and in other areas with high human activity. The specific areas that require this protection may vary across snowy plover life history stages.

## Introduction

Habitat use has important consequences for avian reproductive success and survival (Matessi and Bogliani 1999; Doligez et al. 2002; Sergio et al. 2009). Birds should use breeding areas that maximize access to resources (Sergio and Newton 2003; Preston and Rotenberry 2006; Crampton et al. 2011) while minimizing predation risk to eggs, young, and adults (Ricklefs 1969; Martin and Roper 1988; Powell et al. 2002; Nguyen et al. 2003). In seasonal and heterogeneous environments, habitat selection most likely occurs on several temporal and spatial scales (Hutto 1985) and, for species with precocial young, habitat requirements of different breeding stages may contribute to the complexity of habitat use. Understanding the factors that affect habitat use is important for management programs and restoration projects that attempt to provide habitat for declining species.

In systems with seasonal changes in resources, or for species that utilize different habitats during different parts of the reproductive cycle, occupancy models that allow for movements within the breeding season may be useful (Betts et al. 2008; Rota et al. 2009; Crampton et al. 2011). For example, Betts et al. (2008) showed that young black-throated blue warblers, *Setophaga caerulescens,* initially occupied sites at random and then moved to more suitable territories once more information on habitat quality was available for the birds. Rota et al. (2009) tested for closure (no movement between sampling periods) between two sets of breeding bird surveys. The closure hypothesis was rejected for most species observed. These results indicate that for many avian species, habitat use is not static, and instead occupancy is likely to change throughout the breeding season. The factors that cause birds to move among sites may not be obvious. Betts et al. (2008) suggested that there may be a lack of information available to birds early in the breeding season, so birds adjust their location accordingly as information becomes available. Models that allow for site colonization (transition from an unoccupied site to an occupied site) and extinction (transition from an occupied site to an unoccupied site) have the potential to pinpoint spatial and temporal variations in the landscape that affect apparent movement (MacKenzie et al. 2003). For example, some ground-nesting shorebird species may attempt to minimize predation risk during incubation by choosing cryptic nesting areas with lower risk of predation (Winton et al. 2000; Colwell et al. 2005; Hood and Dinsmore 2007). If chicks are precocial, adults may attempt to move them to foraging areas that will have high food availability during brood-rearing (Cohen et al. 2009; McIntyre and Heath 2011). In a species not bound to its nesting territory during the brood-rearing period, a multi-season occupancy model may provide more information about habitat use in each reproductive stage.

**Figure 1:**
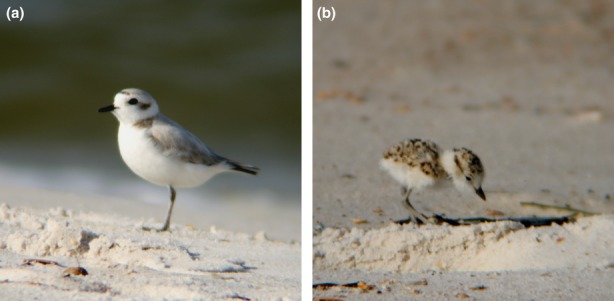
Adult snowy plover *Charadrius nivosus* (a), and precocial snowy plover young (b) on a coastal barrier island along the Florida Panhandle.

Snowy plovers *Charadrius nivosus* (Fig. 1a, b) are territorial, ground-nesting, precocial shorebirds that nest on beaches along the Pacific and Gulf coasts and the interior flats of North America (Page et al. 1995). Snowy plovers are listed as threatened by the state of Florida (Wood 1989) and Pacific coast populations are federally listed in the United States as threatened (Federal Register 1993). Population declines and subsequent listings have been attributed to increased human development and recreational activities in the snowy plovers' breeding and wintering grounds (Gore and Chase 1989; Federal Register 1993). Along the Florida Panhandle, snowy plovers are year-round residents and their annual cycle consists of wintering, pre-breeding (territory establishment), nesting (egg-laying and incubation), and brood-rearing. Pairs may nest again after a failure and pairs that successfully breed early in the season may make a second breeding attempt. Breeding snowy plovers have a patchy distribution along the Florida Panhandle (Lott 2009) and suitable habitat may be limiting. Furthermore, human activities on Panhandle beaches change over the course of the plover breeding season, from relatively few visitors in the winter to thousands of beach goers during the spring and summer. Increased human disturbance may cause plovers to fail or may limit access to suitable breeding areas (Lafferty et al. 2006; Yasué and Dearden 2006; Weston and Elgar 2007).

Our objective was to determine the factors that affect snowy plover site occupancy. We hypothesized that seasonal changes in human disturbance would influence site occupancy; specifically, plovers would avoid high human disturbance. Also, we hypothesized that stage-specific snowy plover habitat requirements would influence site occupancy over the course of the breeding season. We evaluated several habitat features at two different spatial scales. We predicted that small-scale, land cover characteristics, such as beach debris and vegetation between dunes, would affect site use during nesting and that large-scale landform characteristics, such as access to low-energy bayside flats or pools, would influence site use during the mobile brood-rearing stage. We used multi-season occupancy models to test these hypotheses.

## Methods

### Study area

**Figure 2:**
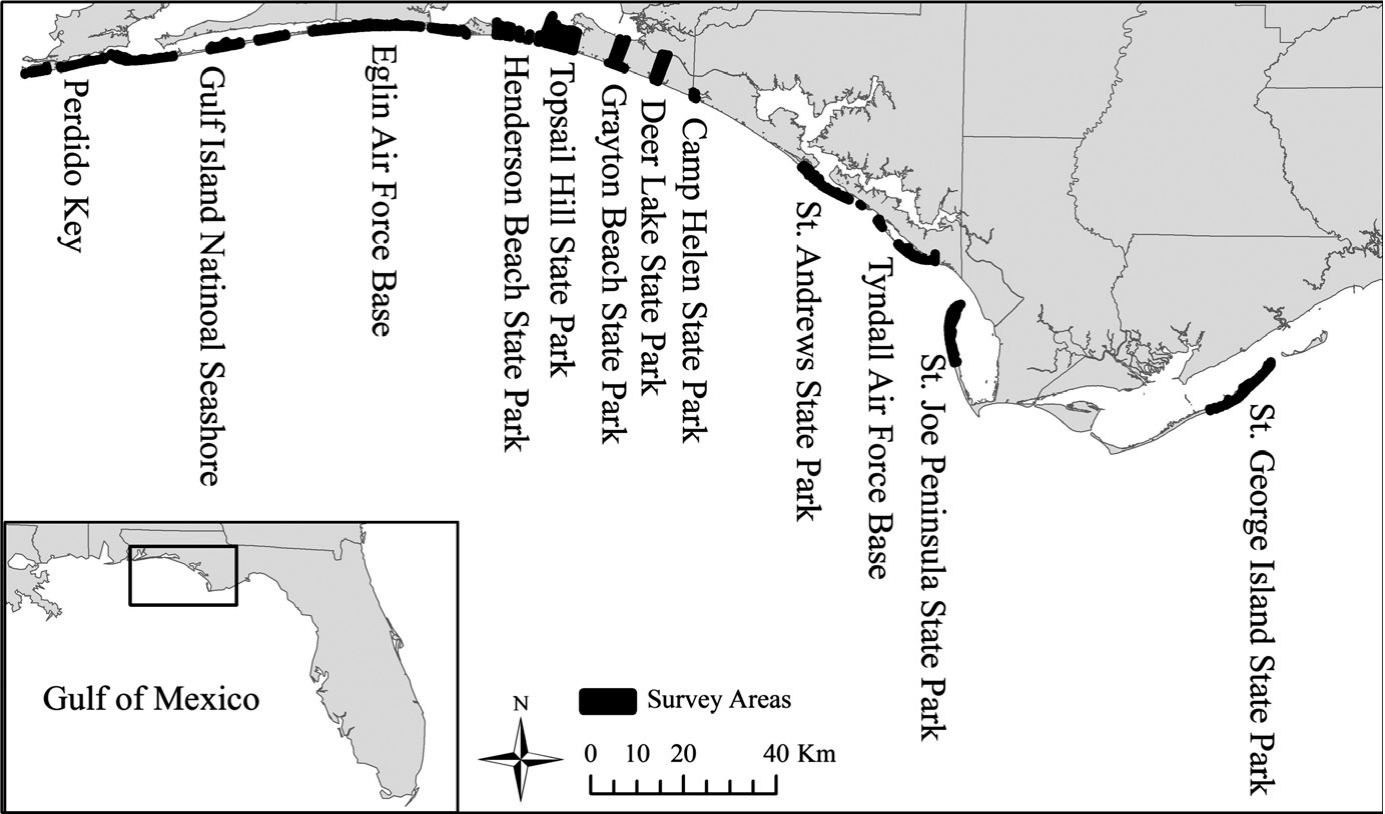
The coastal area of the Florida Panhandle, United States. Black denotes the area where we studied Snowy Plover site occupancy during the 2009 and 2010 breeding seasons (January–July). The study area was divided into 200-m sites running perpendicular to the shore line. We used a stratified-random approach to select 303 non-neighboring sites where we collected information on plover presence, human activity, and beach characteristics.

The Florida Panhandle's barrier islands and coastal areas have been highly developed for human use, except for protected state and federal lands such as Florida State Parks, Department of Defense properties, and National Seashores. Adjacent to these property types, condominiums, vacation houses, and hotels line the beaches just behind the primary dunes. Roads run along the center of most islands and numerous parking areas allow pedestrian access to the beaches. Our study area represented approximately half of sandy beach shoreline of the Florida Panhandle (165 km of 330 km) including coastal areas in Escambia (Perdido Key State Park and Gulf Islands National Seashore [Perdido Key and Ft. Pickens Units]), Santa Rosa (Santa Rosa Island), Okaloosa (Eglin Air Force Base, Ft. Walton Beach, Destin, Henderson Beach State Park), Walton (Topsail Hill State Park, Grayton Beach State Park, Deer Lake State Park), Bay (Camp Helen State Park, St. Andrews State Park, Tyndall Air Force Base), Gulf (St. Joseph Peninsula State Park), and Franklin (St. George Island State Park) counties along the Florida Panhandle (Fig. 2).

### Snowy plovers and human activity

We collected information on plover occupancy, human disturbance, and habitat features from January to July 2009 and 2010. Before sampling, we used aerial images of the Florida Panhandle to divide coastal areas into 200-m-wide, shore-perpendicular sites that stretched from the beach front to the bayside of the barrier island or to the closest major barrier to foot traffic for humans or snowy plover chicks (e.g., a large building or thick vegetation). We used 200-m-wide sites because previous research estimated 100 m for nearest neighbor distances between snowy plover nests (Page et al. 1995; Powell 2001). Thus, occupied sites were likely to have only a single pair. Also, coastal areas can be extremely variable and measurements within 200 m were adequately described beach characteristics. We selected sites using a stratified-random approach to ensure that we sampled sites both in protected (not developed for human use) areas and areas of high human use. We did not select sites that had neighboring sites already sampled. We sampled 101 and 243 sites, respectively, in 2009 and 2010.

We categorized the snowy plover breeding season into three primary sampling periods based on population-level breeding phenology. We considered January through mid-March as “pre-breeding,” when plovers formed loose flocks, pairs engaged in courtship behavior, and females acquired resources for egg formation. There were few nests (<5% annual total) on the Florida Panhandle before mid-March (Himes et al. 2006; Lamonte et al. 2006). We considered mid-March through mid-May to be the nesting period, when plovers paired, established territories, laid eggs, and incubated. During our study, we did not observe any broods on sites during the nesting periods, although some hatching begins before mid-May (Himes et al. 2006; Lamonte et al. 2006). Previous research has shown that habitat features such as beach substrate (e.g., coarse sands or shell) and debris (e.g., driftwood or wrack) (Page et al. 1985; Gore and Chase 1989; Winton et al. 2000; Powell 2001; Hood and Dinsmore 2007; Colwell et al. 2011), distance to dense vegetation (Muir and Colwell 2010), or higher elevation locations that reduce flooding risk during spring storms (Himes et al. 2006) to be predictors of snowy plover success during the nesting phase. Mid-May through July was considered the “brood-rearing” period, when self-feeding, precocial chicks were being brooded or defended by one or more parent. Although most (>75%) pairs were brood-rearing, some pairs also re-nested in this period (Himes et al. 2006; Lamonte et al. 2006). Brood-rearing adults lead chicks to areas of presumably high food availability, like ephemeral pools or the bayside of barrier islands (Loegering and Fraser 1995; Elias et al. 2000), to increase the chances of foraging success (Kosztolányi et al. 2007; Kuwae 2007). Each site was visited for three consecutive days within each of three primary sampling periods: pre-breeding, nesting, and brood-rearing for a total of nine visits each year. If we observed an adult snowy plover within a site on one or more visits during a 3-day sampling period, we considered that site occupied for that primary sampling period.

We measured human disturbance by counting human footprints on a raked-smooth surface of the beach (Engeman and Allen 2000). We raked a 1-m-wide transect from the water to the dune toe on the first day of a primary sampling period and counted the number of tracks the next day (at least 12 h after raking). Then, we re-raked the transect and counted footprints again on the third day of sampling. We divided the footprint count by the transect length and exposure time (number of hours since we raked the transect). Occasionally, high winds or rain destroyed evidence of footprints. In this case, transect exposure time was estimated by the number of hours since the weather event. We averaged the human tracks m^−1^h^−1^ for each primary sampling period. On beaches where raked transects were obliterated by footprints because of high human traffic, we estimated the minimum number of humans walking through the point as 15 human tracks m^−1^h^−1^. This estimate was likely conservative in many cases, as many more than 15 people may have crossed the transect. We validated the human traffic index with the help of a non-partial volunteer who counted the number of humans crossing the smoothed transect for 1 h (*n* = 15). We compared the count of people to the number of human tracks and found that they were the same (min = 3, max = 32).

### Land cover and landform characteristics

We recorded information on land cover characteristics such as interdune vegetation, sand color, sand size, sand sorting, and beach debris. Sites were categorized into one of two categories depending on their interdune vegetation. Beaches where sea oats, *Uniola paniculata,* or shrubby vegetation covered >30% of the interdune area, creating a limited amount of open sand, were categorized as vegetated interdune. Beaches that had patches of vegetation on dunes and large open areas between vegetation were categorized as “open.” The amount of beach debris (shells, asphalt fragments, and dead vegetation) was estimated by tallying the number shells, dead vegetation, or other detritus ≥1 cm intersecting 4, 25-m-long and 1-cm-wide transects in the shape of a “+”.

We measured sand size (m) and sorting (d) from 20-mL samples collected at the toe of the primary dune. We washed samples with distilled water and let them dry for at least 72 h. We weighed the samples and then shook the sand with a sieve shaker (Gilson Company, model SS-15) for 15 min through 6 (−2ф, −1ф, 1ф, 2ф, 2ф, and 4ф) sieves (Folk 1974). The individual size classes were re-weighed to 0.01 g to ensure that all (± 2%) of the sand was recovered from the sieves. We calculated sand size and sorting according to Folk and Ward (1957). We also categorized sand color into three categories: light, medium, and dark (see Webber 2011).

At each site, we recorded beach width and slope, dune height, slope, and length; elevation at the dune toe; and access to low-energy foraging areas as landform characteristics. We measured beach width as the distance between high tide and dune toe. Beach slope was calculated as the percent slope of beach 1.5 m above the high tide (Emery 1961). We measured dune height as the elevation difference between the toe and crest of the dune. Dune slope was the average percent slope on the face of the dune. Dune length was the length of the dune face, parallel to the shore. Elevation was measured at the dune toe with a barometric altimeter that was calibrated at sea level less than 10 min prior to measurement. We recorded as “present” or “absent” snowy plover potential access to low-energy foraging areas such as the island's bayside, a lake, or permanent pool. The most common obstructions to access were dense shrubby vegetation, and man-made obstacles like buildings or walls. We did not directly measure prey availability. Techniques for invertebrate sampling were labor intensive and would have limited the spatial scale of our research. We attempted to use readily measurable site characteristics that may enable managers to identify different suitability of sites at a later date.

### Data analysis

We sampled 40 sites in both years to examine year effects on human activity, land cover, and landform variables. We randomly removed information from one of the years (2009 or 2010) for each of these 40 sites for the occupancy analysis. In addition, one site was missing human disturbance data. The dataset used for occupancy analyses included information from 77 sites sampled in 2009 and 226 sampled in 2010.

We compared single-season occupancy models (no movement) and multi-season occupancy models that accounted for colonization and extinction between the three primary sampling periods (pre-breeding, incubation, and brood-rearing) to test the hypothesis that birds moved between sampling periods. We found support for movement between sampling periods, so we subsequently used multi-season occupancy analysis (MacKenzie et al. 2003) in the program PRESENCE (Hines 2006) to estimate the initial occupancy for each site as well as the subsequent colonization and extinction rates from pre-breeding to nesting and from nesting to brood-rearing. These models assume that plovers did not immigrate or emigrate from a site within the three consecutive visits within a sampling period (i.e., closure), but models allowed for movement between the primary sampling periods. Models based on maximum likelihoods estimated occupancy (ψ), colonization (γ), extinction (ε), and detection probability (*P*) for each site. Initial occupancy (during the pre-breeding season) was calculated and occupancy estimates for nesting and brood-rearing stages were inferred based on colonization and extinction rates between pre-breeding and nesting (γ_1_ and ε_1_) and between nesting and brood-rearing (γ_2_ and ε_2_). We included year as a covariate for detection because the amount of time we spent on a site differed between years. In 2009, we remained on a site for 1 h during each visits and in 2010, the amount of time we sampled a site varied from 5 min to 1 h. We compared model fit using Akaike's Information Criterion adjusted for small sample size (AICc).

We ran pair-wise Spearman correlation analyses for human disturbance, land cover, and landform variables to check for multicollinearity in predictors. For any pair of variables with *r* ≥ |0.70|, we selected the variable with a higher likelihood of affecting snowy plover site use based on findings of previous research. Predictor variables were normalized before analysis. Variable estimates that changed over time were used to predict occupancy for the period when they were measured. For example, human tracks in the pre-breeding sampling period was used to explain initial occupancy and human tracks sampled during the brood-rearing sampling period was used to explain colonization and extinction between nesting and brood-rearing. Variables with estimates that did not change over time, such as vegetation density, were given the same value for all stages.

We used a two-step process to evaluate factors that affect snowy plover occupancy. In the first stage, we used an exploratory approach to build models that explained each model parameter (ψ, γ_1_, γ_2_, ε_1_, and ε_2_). We started with a global set of predictors and used a backwards step-wise selection process where we removed the variable with the lowest absolute value of its parameter estimate divided by its standard error (|β/SE|) until the AICc increased with the removal of the variable with the lowest explanatory power (Zar 1999; Pagano and Arnold 2009). We used an intercept-only model for the remaining parameters in the multi-season occupancy model (Doherty et al. 2012). We considered models with the lowest AICc to be the most parsimonious for each model parameter.

We grouped the predictors from the top models for ψ, γ_1_, γ_2_, ε_1_, and ε_2_ into three categories representing our hypotheses: 1) human disturbance (human tracks m^−1^ h^−1^), 2) land cover (interdune vegetation, debris, sand color, sand size, and sorting), and 3) beach landform (beach width, access to wet foraging areas, elevation, and dune height, slope, and length). We evaluated the evidence for each hypothesis by building multi-season occupancy models that included the variables from each category in each occupancy event. We calculated model averaged parameter estimates based on the models that made 100% of the weight in the hypothesis model comparison (Anderson 2008). We reported 85% confidence intervals for parameter estimates (Arnold 2010). Descriptive statistics were reported as mean (SD).

## Results

Models that included colonization and extinction between primary sampling periods had more support than models that assumed closure (no movement) across the study period (movement model weight = 1.0, non-movement model weight = 0.0). Of the 303 sites, 75 had plovers observed in the pre-breeding period, 118 had plovers in the nesting period, and 147 had plovers in the brood-rearing period. Overall, occupancy increased throughout the season as birds became territorial and moved to brood-rearing foraging areas.

Forty-eight sites had some human development, which was comprised of residences or other structures. During the pre-breeding period, human disturbance averaged 0.018 (0.04) human tracks m^−1^ h^−1^. Human disturbance significantly increased (paired *t* = 3.26, *P* = 0.001) to 0.052 (0.19) human tracks m^−1^ h^−1^ during the nesting period and remained high during the brood-rearing period, 0.055 (0.20) human tracks m^−1^ h^−1^. Human disturbance was lower in 2010 than in 2009 (Wilcoxon *z* = 3.12, *P* = 0.0018), most likely because of the Deepwater Horizon oil spill that occurred on 20-April-2010. The threat of beach closures and swimming restrictions presumably reduced beach recreation. However, snowy plover response to humans did not depend on year (no human × year interaction effect).

Land cover and landform characteristics varied across the Florida Panhandle. Interdune areas were classified as vegetated at 112 (37%) sites and open at 191 (63%) sites. Dark sand was predominant at 71 (23.4%) sites, medium sand at 181 (59.5%), and light sand at 52 (17.1%) of the sites. Debris averaged 0.82 (0.84) objects m^−1^, sand size was 1.61 (0.30) or “fine sand to medium sand” and sorting averaged 0.31 (0.13) or “well sorted to very well sorted” over the course of the study. Average beach width over the course of the study was 43.9 m (26.9 m) during the pre-breeding period, 40.9 m (26.1 m) in the nesting period, and 44.1 m (24.9 m) during the brood-rearing period. The slope above the high tide decreased from January to July, with a beach slope above the high tide mark of 3.4% (3.4%) during pre-breeding, 2.47% (3.22%) during the nesting period, and 0.9% (3.2%) during the brood-rearing period. Dune height averaged 1.41 m (1.02 m), dune slope was 43.14% (32.57%), dune length averaged 85.98 m (128.01 m). Elevation averaged 1.63 m (0.52 m). Access to low-energy foraging areas (typically access to the bayside) was available on 102 (33.6%) of the sites.

**Figure 3:**
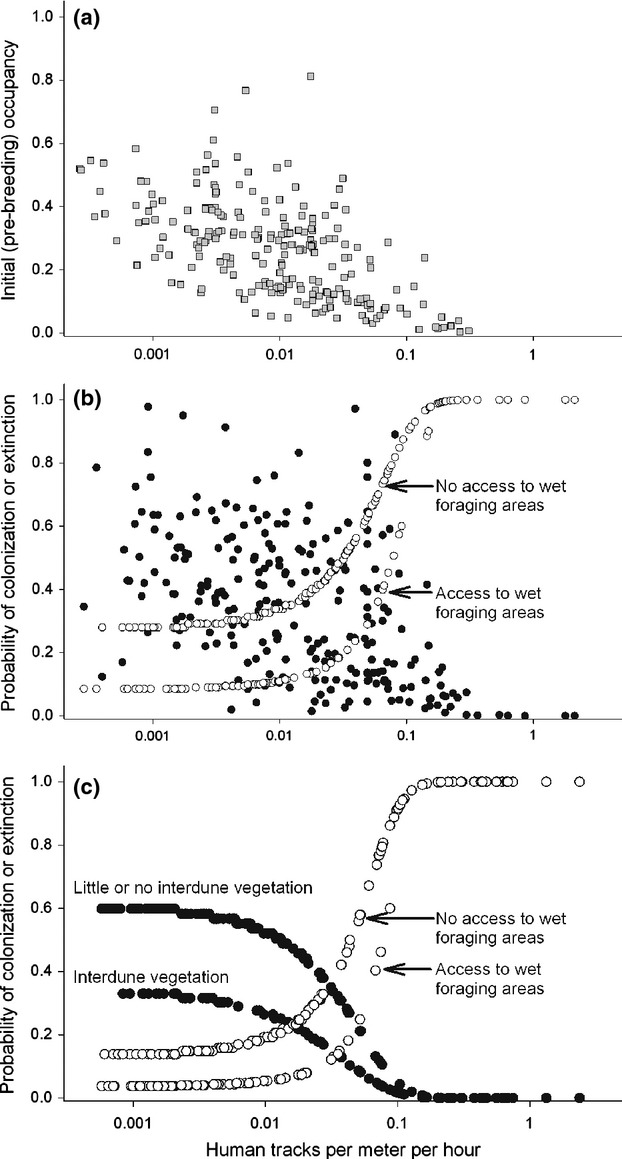
Relationships between human disturbance (tracks m^−1^h^−1^) and predicted site occupancy during the pre-breeding period (a), predicted site colonization and extinction between the pre-breeding and nesting periods (b), and predicted site colonization and extinction between the nesting and brood-rearing periods (c) for snowy plovers on the Florida Panhandle in 2009 and 2010 during the breeding season (January–July). Solid circles represent site colonization (transition from unoccupied to occupied) and empty circles represented site extinction (transition from occupied to unoccupied). Predicted values were estimated from the top multi-season occupancy model. Multiple continuous predictors (see Table 1) were used to calculate pre-breeding site occupancy and site colonization between pre-breeding and nesting, making estimates look scattered. Estimates for extinction between pre-breeding and nesting, and colonization and extinction between nesting and brood-rearing were based on human disturbance and one other dichotomous variable (interdune vegetation or access to wet foraging areas), creating a linear appearance.

During the model building phase of our analysis, we found support for including human disturbance in all occupancy parameters (ψ, γ_1_, ε_1_, γ_2_, and ε_2_). High human disturbance was negatively associated with initial occupancy and both colonization events and positively associated with site extinction (Table 1), indicating that human disturbance negatively impacted snowy plover habitat use during all stages (Fig. 3). Plovers did not use sites with higher human disturbance during pre-breeding, plovers were not likely to colonize sites with higher human disturbance, and sites where human disturbance increased were likely to go extinct. Land cover characteristics such as interdune vegetation were negatively associated with ψ, γ_1_, and γ_2_ (Table 1). The amount of debris had a positive effect on γ_1_, indicating that plovers moved into areas with more debris when selecting nesting sites (Table 1). There was no evidence for including sand size, sorting, or color in predicting plover site occupancy. Dune height, slope, and length predicted ψ, with areas of higher, shorter, flatter dunes more likely to be occupied. Dune slope and elevation predicted γ_1_, suggesting that plovers moved to higher sites when selecting nest areas. Access to low-energy foraging areas like the bayside of the island was negatively associated with ε_1_ and ε_2_ (Fig. 3), indicating that sites with foraging areas were less likely to have plovers move away. There was no evidence that beach width affected plover site occupancy.

**Table 1:** Model averaged parameter estimates, standard error (SE), and 85% confidence limits for normalized variables within each occupancy event. Superscripts represent stage-specific human disturbance estimates, PB: pre-breeding, N: Nesting, BR: brood-rearing

	β	SE	Lower CI	Higher CI
ψ	−0.632	0.199	−0.918	−0.346
Humans^PB^	−0.509	0.286	−0.921	−0.097
Veg	−1.043	0.374	−1.582	−0.504
D_ht	0.622	0.195	0.342	0.903
D_slp	−0.186	0.113	−0.349	−0.023
D_len	−0.295	0.172	−0.542	−0.048
γ_1_	−0.310	0.277	−0.709	0.090
Humans^N^	−1.145	0.684	−2.130	−0.160
Veg	−1.216	0.417	−1.817	−0.615
Debris	0.528	0.242	0.179	0.876
Elevation	−0.667	0.285	−1.078	−0.256
D_slp	0.413	0.208	0.114	0.712
ε_1_	0.439	0.770	−0.670	1.547
Humans^N^	5.210	3.343	0.395	10.024
Bay	−1.141	0.809	−2.306	0.023
γ_2_	−1.265	0.661	−2.217	−0.313
Humans^BR^	−6.097	2.765	−10.079	−2.116
Veg	−1.060	0.470	−1.736	−0.383
ε_2_	0.067	0.741	−1.000	1.135
Humans^BR^	7.067	3.229	2.418	11.716
Bay	−0.897	0.621	−1.791	−0.003

Humans: number of tracks m^−1^h^−1^, Veg: interdune vegetation present, D_ht: Dune height, D_slp: Dune slope, D_len: Dune length, Debris: debris on beach m^−1^, Elevation: elevation at dune toe, Bay: access to bayside of barrier island, lakes, or permanent pools.

The multi-season occupancy model that contained predictors from all three hypotheses, human disturbance, land cover, and landform, had the lowest AICc (Table 2). The next highest model included human disturbance and landform characteristics, but was >2 ΔAICc from the top model and had a low weight, suggesting a model that included seasonal changes in human disturbance and stage-specific habitat features at the land cover and landform scale had the most support.

**Table 2:** Model comparison of human disturbance, land cover, and landform hypotheses to explain snowy plover site occupancy during pre-breeding (ψ), site colonization between pre-breeding and nesting (γ_1_), site extinction between pre-breeding and nesting (ε_1_), site colonization between nesting and brood-rearing (γ_2_), site extinction between nesting and brood-rearing (ε_2_), and detection (p) on Florida Panhandle beaches in 2009 and 2010. ΔAICc is the difference in AICc score from the top model, *w* is the model weight, and *K* is the number of parameters included within the model. Superscripts represent stage-specific human disturbance estimates, PB: pre-breeding, N: Nesting, BR: brood-rearing. For variable names, see Table 1

Hypotheses	Model	Δ AICc	*w*	*K*
Disturbance + Land Cover + Landform	ψ(Humans^PB^ + Veg + D_ht + D_slp + D_len), γ_1_ (Humans^N^ + Veg + Debris + Elevation + D_slp), ε_1_(Humans^N^ + Bay), γ_2_(Humans^BR^ + Veg), ε_2_(Humans^BR^ + Bay), p(Year)	0	0.9997	22
Disturbance + Landform	ψ(Humans^PB^ + D_ht + D_slp + D_len), γ_1_ (Humans^N^ + Elevation + D_slp), ε_1_(Humans^N^ + Bay), γ_2_(Humans^BR^), ε_2_(Humans^BR^ + Bay), p (Year)	16.55	0.0003	18
Disturbance + Land Cover	ψ(Humans^PB^ + Veg), γ_1_ (Humans^N^ + Veg + Debris), ε_1_(Humans^N^), γ2(Humans^BR^ + Veg), ε_2_(Humans^BR^), p (Year)	18.89	0.0001	16
Disturbance	ψ(Humans^PB^), γ_1_ (Humans^N^), ε_1_(Humans^N^), γ_2_(Humans^BR^), ε_2_(Humans^BR^), p (Year)	38.69	0	12
Land Cover + Landform	ψ(Veg + D_ht + D_slp + D_len), γ_1_ (Veg + Debris + Elevation + D_slp), ε_1_(Bay), γ_2_(Veg), ε_2_(Bay), p (Year)	39.63	0	17
Land Cover	ψ(Veg), γ_1_(Veg + Debris), ε_1_, γ_2_(Veg), ε_2_(.), p (Year)	62.50	0	13
Landform	ψ(D_ht + D_slp +D_len), γ_1_(Elevation + D_slp), ε_1_(Bay), γ_2_(.), ε_2_(Bay), p (Year)	77.88	0	11
Intercept-only	ψ(.), γ_1_(.), ε_1_(.), γ_2_(.), ε_2_(.),p (.)	106.76	0	4

Lowest AICc = 2205.47.

## Discussion

On Florida's panhandle coast, snowy plovers moved during the course of the breeding season to adjust to changing levels of human disturbance and satisfy changing resource needs from pre-breeding to nesting to brood-rearing. The use of multi-season occupancy analysis was a useful tool for identifying habitat parameters that influence habitat use. Stage-specific resource requirements may be an overlooked aspect of information in developing management plans for protected species. In particular, species not bound to nesting areas, like some shorebirds with precocial young, may move to habitats where young birds learn to forage.

Human disturbance played a strong role in predicting snowy plover habitat use throughout the study. Humans may be perceived as predators to adults, eggs, or young (Flemming et al. 1988; Verhulst et al. 2001; Beale and Monaghan 2004; Burger et al. 2007; Weston and Elgar 2007). High levels of human traffic may increase the chances that eggs are trampled (Weston et al. 2012). Human traffic also may disturb plover foraging (Burger 1994), as plovers frequently feed on terrestrial insects that cluster around the wrack line where people prefer to walk. Foraging plovers interrupted by humans stopped foraging, moved away from the wrack, and stood until the disturbance had passed. If a bird spends too much time avoiding disturbance, it may not be able to dedicate the time necessary to hunt invertebrates, regardless of the amount of food available (Weston and Elgar 2005). Regardless of the mechanism, decreased use of sites with higher human activity limits snowy plover breeding distributions and may constrain plover populations (Yasué and Dearden 2006).

An effective tool for reducing the impact of human disturbance is the use of signage and symbolic fencing to keep beach recreationists away from nesting areas (Weston et al. 2012). This technique combines the use of signs to indicate the presence of nesting birds and string, tied between posts, to section off a part of the beach for shorebird nesting. It has been successful in the past in reducing the impacts of disturbance on snowy plovers in California (Lafferty et al. 2006; Wilson and Colwell 2010) and piping plovers in New York (Doherty and Heath 2011). One area in this study (Deer Lake State Park) had a large area of symbolic fencing that restricted pedestrians to areas near the high tide line. This was an area where high human traffic coincided with snowy plover nesting. These sites had pre-breeding disturbance levels twice as high as the average snowy plover occupied sites, and the brood-rearing disturbance levels were higher than the average beach without snowy plovers. Nonetheless, several pairs of plovers nested at this site in 2009 and 2010, at least one of which successfully hatched chicks each year. While the symbolic fencing did not decrease human traffic, it may have restricted its effects to a localized area that the birds could choose to avoid.

Interdune vegetation was negatively associated with plover habitat use. Snowy plover brood avoidance of vegetation may negatively coincide with use of foraging areas that have wet sand (Loegering and Fraser 1995; Elias et al. 2000; Fraser et al. 2005) as wet sand is not conducive for vegetation growth. Alternatively, vegetated areas may have higher predator densities or vegetation may affect an incubating plover's ability to detect predators and successfully perform a “broken-wing” display. The display typically attracts potential predators to the adult who feigns injury and leads the predator away from the nest. For this ploy to be effective, a nesting adult may need to identify a threat early (by line of sight). Muir and Colwell (2010) found that western snowy plovers selected nesting habitat that was free of dense vegetation in a radius that was similar to their flushing distance when a human approached. In dense vegetation, predators may be more difficult to spot, and the adults may have more difficulty maneuvering through dense vegetation to a point where the predator can easily notice the display. At times, we observed plover broods hiding in vegetation clumps in response to the adults' alarms. It is possible that dense interdune vegetation prevents early detection of predators, but some vegetation is advantageous for cover. For example, artificial shelters increased survival rates of Hooded Plover *Thinornis rubricollis* broods, indicating that cover may be an important part of reproductive success (Maguire et al. 2011).

The amount of debris on the beachfront was positively associated with snowy plover presence during the nesting period. Other studies have found that a higher percentage of shell of pebble cover is positively associated with other Charadriiformes habitat use (Winton et al. 2000; Nguyen et al. 2003; Colwell et al. 2005; Hood and Dinsmore 2007). A nest placed among debris on the beach may be less likely to be depredated, as shells and vegetation act as camouflage for the nest itself.

Dune structure and access to bay each had an effect on snowy plover occupancy. In the pre-breeding period, birds were positively associated with high, narrow dunes that were gently sloped. Dunes that are narrow in length or gently sloped allow birds to easily walk behind the dunes during times of storms when the beachfront may not be as safe or to escape heavy recreation on the beach front; however, dune slope during this period had the smallest estimate within the occupancy model, followed by dune length as the second smallest estimate, so the effect may be smaller relative to other factors. Higher elevation of dune toe also was positively associated with initial snowy plover occupancy. High elevation may decrease the chance of nests on the beachfront being washed away in storms (Himes et al. 2006). The positive association with plovers and bay access during the nesting and brood-rearing stage is consistent with research that shows bay areas typically have high invertebrate density and provide important foraging areas for young plovers and adults (Cohen et al. 2009).

Snowy plover habitat requirements may be more specific during the nesting and brood-rearing than during the wintering or pre-breeding stages. Although during all stages, they select habitat with lower human disturbance and vegetation densities, they tend to colonize areas for breeding that have higher amounts of debris, dunes that are tall in height and short in length, beaches with higher elevation, and access to wet foraging areas. Increasing coastal development counteracts most of these habitat characteristics by providing more access areas for beach goers, increasing beach raking, which decreases debris and substrate for insect prey, and increasing structures or busy roads, which may restrict access to the bayside of a barrier island. Breeding snowy plovers would likely benefit from management that provides connectivity among beachfront, dune, and wet foraging habitats to provide a range of food resources, as well as refugia from predators and human disturbance. Given the shifting resource needs documented here, management actions such as string fencing to reduce human disturbance may need to be in different areas across a breeding season, protecting nesting areas during incubation and then chick foraging areas during the brood-rearing period. Finally, this study supports the idea that birds make adjustments to habitat use depending on current conditions (disturbance) and resource needs (foraging areas) that would optimize reproductive potential.
